# Comprehensive Validation of the TrAI4Nel Simulator for Nelore Artificial Insemination Training: A Controlled Study

**DOI:** 10.3390/ani15202982

**Published:** 2025-10-15

**Authors:** Heitor Azuaga-Filho, Alexandre Santos, Bruno Colaço, Rita Payan-Carreira

**Affiliations:** 1 Instituto Federal de Educação, Ciência e Tecnologia de Mato Grosso—Campus Cáceres, Cáceres 78201-382, MT, Brazil; heitor.filho@ifmt.edu.br (H.A.-F.); alexandre.santos@ifmt.edu.br (A.S.); 2 Veterinary and Animal Research Centre (CECAV), University of Trás-os-Montes and Alto Douro (UTAD), 5000-801 Vila Real, Portugal; bcolaco@utad.pt; 3 Associate Laboratory for Animal and Veterinary Sciences (AL4AnimalS), University of Trás-os-Montes and Alto Douro (UTAD), 5000-801 Vila Real, Portugal; 4 Comprehensive Health Research Centre (CHRC), Universidade de Évora, 7004-516 Évora, Portugal; 5 Escola de Ciências e Tecnologia, Departmento de Medicina Veterinaria, Universidade de Évora, 7004-516 Évora, Portugal

**Keywords:** bovine AI training, *Bos indicus*, simulator’s validity, training effectiveness, training success, simulation education

## Abstract

**Simple Summary:**

Artificial insemination is a crucial technique for cattle breeding that helps farmers improve their herds, but training people to perform this procedure has traditionally required using abattoir specimens or live cattle, which raises animal welfare concerns and can be stressful for trainees. Most training simulators are designed for European cattle breeds, but many regions rely on zebu cattle (like Nelore cattle in Brazil), which have different anatomy. In this study, a new training simulator specifically designed for zebu cattle artificial insemination called TrAI4Nel is tested. Authors compared training outcomes between students who learned using traditional methods with abattoir specimens versus those who used the new simulator. The study involved 61 trainees and 14 experts who evaluated the simulator’s effectiveness. Results showed that students trained with the simulator performed significantly better when working with live animals, achieving a 79% success rate compared to 53% for traditionally trained students. The simulator-trained students also felt more confident and less anxious. The simulator accurately replicated the anatomy and procedures needed for zebu cattle, making it an excellent training tool that improves learning outcomes while protecting animal welfare. This technology can help regions dependent on zebu cattle improve their breeding programs more effectively and humanely.

**Abstract:**

Effective bovine artificial insemination (AI) training requires balancing technical skill development with animal welfare considerations. Commercial simulators typically replicate *Bos taurus* anatomy, limiting utility in regions where *Bos indicus* breeds predominate. This study validates the TrAI4Nel simulator, customized for Nelore cattle AI training. Validation employed a multi-dimensional framework encompassing face, physical, content, construct, and concurrent validity, plus usability and training effectiveness assessments. Of the 85 participants in standardized AI technician courses who were randomly allocated to control (abattoir specimen-based) and experimental (simulator-integrated) groups, 61 provided feedback about TrAI4Nel (19 in the control group and 42 in the experimental group). The simulator was also independently evaluated by 14 AI experts. Trainees rated the simulator highly for anatomical realism and procedural consistency. Compared with abattoir specimens, TrAI4Nel significantly enhanced skill transfer to live animals, particularly cervical pipette navigation and semen deposition identification. The simulator increased trainee confidence, reduced anxiety, and improved perceived preparedness. Performance assessments demonstrated simulator-trained participants achieved significantly higher success rates (78.6%) versus controls (52.6%; *p* = 0.043), without prolonging completion times. Qualitative feedback emphasized the simulator’s pedagogical value in enhancing anatomical comprehension, skill acquisition, and learner autonomy while supporting animal welfare. Training sequence analysis indicated biological specimen exposure before simulator use may optimize learning efficiency. These findings validate TrAI4Nel as an effective, ethically sound tool for *Bos indicus* AI training. Simulator integration into curricula provides scalable improvement of reproductive management in zebu-dependent regions.

## 1. Introduction

Effectively training professionals in animal reproduction procedures presents a dual challenge: ensuring adequate development of technical competencies while addressing ethical concerns regarding animal welfare. Traditionally, mastering procedures such as artificial insemination (AI) required extensive hands-on practice with live animals, raising concerns about animal stress and trainees’ initial awkwardness [1,2]. Simulation-based training has emerged as a critical strategy that addresses both ethical considerations and educational needs by providing standardized, repeatable practice opportunities in risk-free environments. Synthetic models and simulators enable students to develop essential tactile skills and spatial awareness, minimizing the risks for animal and student anxiety in the initial learning phases [1,3].

The integration of simulators in veterinary education has demonstrated significant benefits in enhancing student perceptions, satisfaction, and perceived usefulness. Students report positive feedback regarding simulator use, particularly appreciating the opportunity to practice clinical skills repeatedly in controlled settings without time pressure or risk of patient harm, while the flexibility to engage with learning materials at individual pace contributes to higher satisfaction levels compared to conventional teaching methods [1,4]. Meta-analyses indicate that simulation training effectively improves knowledge retention and clinical skill development across varying levels of student experience, demonstrating consistent educational value regardless of prior expertise [5,6,7]. Although simulators are recognized as valuable educational tools that provide safe, flexible, and effective means for acquiring essential clinical competencies, continued research and development of simulation technologies are necessary to expand their application across all domains of veterinary practice [8].

The shift toward competency-based learning paradigms in veterinary and animal sciences education drove the need to reduce and refine the use of animals for training and has accelerated the development and implementation of diverse simulation models [9]. In bovine theriogenology specifically, commercial simulators now exist for training transrectal palpation and artificial insemination [10,11], pregnancy diagnosis [12] or obstetrics procedures in the parturient cow [13]. These high-fidelity models not only enhance technical skills through standardized repetitive practice of real-life replicated situations [5] but can also foster development of professional competencies including confidence, communication, and decision-making when integrated into comprehensive educational frameworks [14,15,16].

Despite their educational value, current bovine reproduction simulators face several limitations. High acquisition costs restrict widespread implementation and individual student access [1]. Commercial simulators are usually expensive, and their repetitive use necessitates regular replacement of components due to wear and tear that can affect their anatomical accuracy over time [17]. To address budget constraints, educators are often encouraged to construct low-fidelity or low-technological models to support basic procedure training, as both high-fidelity and low-fidelity models allowing multiple repetitions can improve students’ proficiency and self-confidence.

From a technical perspective, even advanced simulators struggle to perfectly replicate the complex tactile feedback and tissue consistency encountered in live animals [3], or fail to reproduce the natural variations in animal movement and behavior [10,18]. Educational concerns also exist regarding technique habits specific to simulators that may not translate optimally to live animal procedures, particularly regarding the appropriate pressure applied during transrectal examinations [19] or instrument manipulation [20]. Furthermore, simulation-based training must be carefully balanced with live animal experience to prevent potential overconfidence in abilities developed through standardized scenarios that lack the biological variation encountered in practice [21].

A critical limitation of commercially available bovine AI simulators that has received insufficient attention is their anatomical basis. Current models predominantly represent *Bos taurus* cervical anatomy, creating a significant educational gap for students and professionals working with *Bos indicus* breeds such as Nelore cattle, which are characterized by longer and more tortuous cervical anatomy [11]. This anatomical disparity potentially compromises the training efficacy for practitioners working in regions where *Bos indicus* breeds predominate, including substantial portions of Latin America, Africa, and Asia.

To maximize educational outcomes from simulation training and increase learning success, three dimensions require careful consideration. First, simulator-related aspects, including the acceptability of models by trainees, perceived fidelity, ease of manipulation, and anatomical accuracy. Second, implementation within structured educational frameworks, including proper feedback mechanisms, well-designed scenarios, and debriefing that target the development of good practices. Third, rigorous validation of training outcomes and reporting standards that relate to task assessment quality [7,14]. Most commercially available physical simulators for bovine AI training enable repetitive practice under standardized conditions and safe settings, diminish reliance on live animals, alleviate student anxiety, foster motivation for procedure execution, and provide opportunities to learn from errors without jeopardizing animal welfare [11]. However, despite their widespread use in reputable institutions, most artificial insemination models have only been evaluated through subjective face validity assessments, leaving their educational effectiveness inadequately characterized, particularly considering their significant investment costs.

This study addresses these gaps by testing the hypothesis that the TrAI4Nel simulator [17], specifically designed for training artificial insemination in Nelore cattle, provides superior educational outcomes compared to traditional abattoir-based training methods.

We hypothesized that comprehensive validation across multiple psychometric dimensions would demonstrate the simulator’s effectiveness in enhancing skill acquisition and transfer to live animal procedures. The primary aim was to conduct a rigorous validation of the TrAI4Nel simulator employing multiple methodological approaches, including analysis of self-reported experiences from trainees and experts, and examination of simulator-generated performance metrics (procedure correctness and cervical barrier passage time). The validation framework encompasses five dimensions: face validity (realism assessment), content validity (coverage of essential AI procedure steps), concurrent validity (correlation with real-life performance), predictive validity (perceived transferability of learned skills), and usability (ease and intuitiveness of simulator interaction). The secondary aim was to evaluate the simulator’s practical effectiveness within short training programs for service providers, thereby establishing its efficacy and potential contributions to breeding technician education in regions where Nelore cattle predominate.

## 2. Materials and Methods

The TrAI4Nel simulator [17] was evaluated by participants enrolled in training courses for artificial insemination service providers, individuals with no prior academic training in animals or veterinary sciences. These short courses were conducted at the Artificial Insemination Center of the Federal Institute of Education, Science, and Technology of Mato Grosso (IFMT)—Cáceres Campus—Prof. Olegário Baldo, with live animal training conducted at Ressaca Farm of the Grendene Group. The study was conducted in accordance with general ethical principles and received approval from the Research Ethics Committee of IFMT (Protocol number R3353/21, approved on the 23 November 2021).

### 2.1. Study Design

The TrAI4Nel simulator represents an innovative, anatomically precise training platform customized for artificial insemination (AI) in Nelore cattle (*Bos indicus*) (Supplementary Materials Figure S1), whose development process has been recently described [17]. Developed through advanced 3D modeling and additive manufacturing techniques, this simulator accurately replicates the reproductive tract of Nelore cows, incorporating the uterus, cervix, and ovaries based on precise morphometric data obtained from abattoir-collected specimens. The cervix model features a dual-layer silicone rubber construction designed to effectively reproduce the tactile sensations experienced during pipette navigation through cervical rings, thereby enhancing training realism. To provide objective performance assessment, the simulator integrates electronic components, including a Reed Switch sensor and LED feedback system that delivers real-time guidance on pipette placement accuracy and signals optimal semen deposition zones. Furthermore, an arduino-controlled peristaltic system simulates rectal palpation conditions through inflatable bicycle chambers filled with coolant fluid, closely approximating live-animal handling dynamics [17].

To test and validate the TrAI4Nel simulator, we established three non-overlapping experimental groups, with each group assigned to a distinct pedagogical approach: one classical approach using abattoir specimens (control group) and two experimental approaches (described below). All groups were enrolled in a standardized 3-day training program. Training sessions were organized with a maximum of ten participants per group, with each session exclusively dedicated to a single group. The program consisted of four hours of theoretical instruction covering bovine reproductive anatomy, estrous cycle physiology, physiological changes, frozen semen storage and handling, and proper semen deposition techniques in cattle. This was followed by four hours of hands-on training using ex vivo specimens with or without simulator access prior to training with live animals at *Fazenda Ressaca* (Ressaca Farm). All participants received identical standardized introductions regarding the use of models and simulators for animal welfare considerations, as well as identical theoretical instruction. The hands-on training component varied according to group assignment using a crossover design in the experimental groups.

The control group participants (Gp C) received four hours of training in genital tract manipulation and catheter passage through the cervix using abattoir specimens for four hours, followed by sixteen hours of *in vivo* training, achieving a total hands-on training duration equivalent to that of the experimental groups. The experimental group was divided into two subgroups, both utilizing the simulator and abattoir specimens in reverse sequences. Group 1 (Gp 1) began with four hours of simulator-based hands-on training, progressed to four hours of training with abattoir specimens, and completed twelve hours of *in vivo* training with live cows. Group 2 (Gp 2) initially received four hours of training with abattoir specimens (four hours), followed by four hours simulator training, and concluded with twelve hours of *in vivo* training.

The *in vivo* training was conducted at *Ressaca Farm* using cohorts of cows (25 cows for the control group and 18 cows for each experimental group). Throughout the *in vivo* training period, trainees were allowed a maximum of three minutes per insemination attempt on each cow, the total number of attempts per student restricted by the number of available cows. Upon reaching the correct anatomical position, students were required to summon the supervising instructor for procedure validation. If the insemination pipette could not be successfully advanced through the cervix in the allocated time, the attempt was classified as unsuccessful. For each trainee, every attempt was recorded as successful or unsuccessful, and the time required to achieve successful insemination was documented. Following completion of the *in vivo* training, and after a minimum 5 min interval, trainees underwent individual assessment for certification. The evaluation assessed students’ technical proficiency in the procedure.

At the end of the training program, participants were requested to complete a questionnaire reporting their perception of the simulator compared to the use of abattoir specimens and live animal experiences.

### 2.2. Data Collection and Tools

Data collection was conducted through an online questionnaire specifically designed to evaluate trainees’ perceptions of the cattle artificial insemination (AI) training program (Supplementary Materials Table S1). The questionnaire was developed in Portuguese and distributed electronically via Google Forms. Participation was voluntary, and all participants provided informed consent before accessing the questions. The questionnaire contained 31 questions organized into five sections (Supplementary Materials Table S1):

1. Informed Consent: The informed consent process used a binary response system where affirmative responses (‘Yes’) granted access to the questionnaire, while negative responses (‘No’) triggered automatic survey termination.
2. Personal Information and Prior Knowledge: This section captured participant demographics including gender, age, and educational background, along with professional experience in livestock management and bovine artificial insemination procedures.
3. Training Experience with Biological Models: The first question in this section served as a screening tool to distinguish between experimental and control groups, directing participants to the appropriate subsequent sections. Additionally, it assessed participant perceptions regarding the use of biological reproductive tracts obtained from abattoirs for practical training. Evaluation criteria included perceived realism, ease of manipulation, and educational efficacy for skill acquisition. Control group participants completed the questionnaire at this juncture.
4. Training Experience with the Simulator: Participants in the experimental groups, who received exposure to both the TrAI4Nel simulator and biological models, were asked to evaluate their simulator experience and provide comparative assessments relative to biological reproductive models and live animal procedures. This section assessed perceived realism, procedural consistency, and educational utility of the simulation platform.
5. Final Evaluation and Perceived Competence: This concluding section incorporated comprehensive assessments of participant satisfaction, self-perceived confidence for independent AI performance, and perceived significance of simulation-based training in procedural skill development.

The questionnaire, specifically developed for this study, employs a standard five-levels Likert scaling methodology to measure agreement with key statements, with options ranging from “1—Strongly Disagree” to “5—Strongly Agree.” Other items were multiple-choice, short-answer, or open-ended questions allowing qualitative feedback. This mixed-method approach enabled collection of both quantitative data for statistical analysis and qualitative insights to capture trainees’ opinions and experiences in their own words.

The questionnaire underwent pilot testing with a small group of trainees (n = 5) to ensure clarity, relevance, and internal consistency. Minor adjustments were made based on feedback from this preliminary evaluation.

A condensed, modified version of the questionnaire was administered to experienced AI providers (henceforward named as experts), focusing primarily on simulator-related assessment criteria (Supplementary Materials Table S2).

To document the translation of trainees’ performance to live cows during the final assessment, data were recorded by the instructor (the first author) using Excel spreadsheets, which included information on the outcome of each attempt (success vs. failure, or withdrawal), the final placement of the pipette, and the time required to complete a successful attempt for each cow-trainee pairing. Success was consistently defined as passage of the pipette through the cervix. These records were subsequently used to compare the effect of the simulator on AI technique training outcomes.

### 2.3. Study Population

The training program was offered to participants with diverse educational profiles and varied backgrounds (but no formal training in bovine anatomy) who intended to become bovine artificial insemination service providers. Out of 90 available training positions, 85 were filled (29 in Gp C, 27 in Gp 1, and 29 in Gp 2), with 78 trainees successfully completing the course. The remainder participants either voluntarily withdraw or failed the final examination. All participants were invited to complete the questionnaire about their training experience, regardless of whether they completed the course.

In addition to the trainee groups, fourteen experts with professional experience providing regular bovine artificial insemination services were invited to test the simulator and provide insights into the use of the TrAI4Nel, using selected items from the trainees’ questionnaire that specifically related to the simulator.

### 2.4. Validation Constructs

The TrAI4Nel simulator validation incorporated the following constructs through collective analysis of data from respondents in all the groups:

Face Validity—examines whether the simulator appears, from the user’s perspective at surface level, to reasonably represent the reproductive tract structures manipulated during bovine artificial insemination.

Physical Fidelity—evaluates the degree to which the simulator’s physical characteristics replicate those of the real biological system (e.g., anatomy, texture, or hand movement).

Content Validity—assesses whether the simulator comprehensively covers all essential components and skills within the AI procedure domain.

Construct Validity—determines whether the simulator effectively ensures an accurate representation of skills and knowledge required for successful AI.

Concurrent Validity—examines how well the simulator training outcomes correlate with established training methods administered simultaneously (namely, the abattoir specimens and the living cow).

Supplementary Materials Table S3 identifies the questionnaire items utilized for each validation construct. Specific closed-ended and open-ended questions from the questionnaire were used to gather user feedback and conduct the User Satisfaction analysis, which aimed to capture additional information covering various aspects of satisfaction and usability that influence training acceptance and effectiveness.

For Training Effectiveness, data were extracted from the records of participants’ practical assessments who had responded to the questionnaire (number of attempts until success, successful AI within the first three attempts and global success, average number of attempts).

### 2.5. Statistical Analysis

All analyses were conducted using R statistical software (version 4.3.1), with DHARMa and emmeans packages. Data are presented as means ± standard error for continuous variables and frequencies/percentages for categorical variables. Medians and ranges are reported where appropriate.

Likert scale responses (1–5) were analyzed using descriptive statistics. Face, physical, content, construct, and concurrent validity were assessed through participant responses comparing the TrAI4Nel simulator to abattoir specimens and live animal. Mean scores were computed independently for trainees and experts, with responses categorized as negative (scores 1–2), neutral (score 3), or positive (scores 4–5). Validation thresholds were established at mean scores ≥ 4.0 for all constructs.

Content analysis of open-ended responses employed an inductive approach, allowing thematic categories to emerge from the data. Responses were systematically reviewed to identify recurring themes until data saturation was achieved. Representative quotes were selected to illustrate each category, capturing both positive and negative aspects of simulator experiences.

Success rates and completion times were recorded during training and final assessment phases. Generalized linear models (GLMs) compared training effectiveness between the control group (traditional reproductive tract training; GpC) and experimental groups with different sequences: simulator-first (Gp1) versus reproductive tracts-first (Gp2). Model fit and variance homogeneity were assessed to ensure analytical robustness. Post hoc comparisons used least squares means tests when significant differences were detected.

Statistical significance was set at α = 0.05 for all analyses. Effect sizes were calculated using generalized eta-squared (η^2^G) and interpreted as small (0.01 ≤ η^2^G < 0.06), moderate (0.06 ≤ η^2^G < 0.14), or large (η^2^G ≥ 0.14) to determine the practical significance of observed differences.

## 3. Results

### 3.1. Participant Characteristics and Response Rates

Of the 85 enrolled trainees, 78 successfully completed the course (25 in GpC, 26 in Gp 1, and 27 in Gp 2), yielding an overall completion rate of 92% (86%, 96%, and 93% for GpC, Gp1, and Gp2, respectively). Among course completers, 65 participants responded to the simulator questionnaire. Following exclusion of four control group respondents with prior bovine AI experience, data from 61 questionnaires were analyzed (19 GpC, 19 Gp1, and 23 Gp2), corresponding to a final response rate of 71.8%. Simulator users comprised 68.9% (n = 42) of the analyzed sample.

Males were overrepresented among respondents (68.9%, n = 42) compared to females (31.1%, n = 19) across all trainee groups (GpC: 12 vs. 7; Gp1: 13 vs. 6; Gp2: 17 vs. 6). Trainees’ ages ranged from 16 to 54 years, with Gp1 having the lowest mean age (23.16 ± 1.50 years, median = 20) compared to the GpC (28.32 ± 2.72 years, median = 26) and Gp2 (28.87 ± 2.19 years, median = 27) (Table 1). Expert evaluators (n = 14; 92.4% male) had a mean age of 34.43 ± 11.27 years (median = 29) (Table 1) and averaged 7.77 ± 4.15 years of bovine AI experience (median = 6 years, range: 3–15 years); 64.3% (n = 9) performed bovine AI regularly.

These AI training courses were open to participants regardless of formal background in veterinary medicine or animal husbandry. None of the trainees possessed prior formal education in animal science or veterinary medicine, which is not a prerequisite for enrollment in technical certification programs. Educational backgrounds varied considerably among groups (Table 2). High school education was the most prevalent qualification in both the GpC (78.95%, n = 15) and Gp1 (73.68%, n = 14), whereas Gp2 demonstrated greater educational diversity, with high school (47.83%, n = 11) and completed college education (34.78%, n = 8) being the most common qualifications. Grade school education was represented only in the GpC (5.26%, n = 1). Occupational backgrounds showed equal representation between rural producers/farmers and students (n = 12 each), followed by administrative assistants (n = 9) and unemployed individuals (n = 8). Notably, 32.8% of respondents (n = 20) reported having no prior experience with cattle handling (Table 3).

Experts had a mean of 7.7 ± 1.15 years of experience providing artificial insemination services (range: 3–15; median = 6 years). They either provided sporadic AI services in Nelore cattle (n = 5; 35.7%) or provided them routinely (n = 9; 64.3%).

### 3.2. Validity Assessment

#### 3.2.1. Face Validity

**Figure 1:**
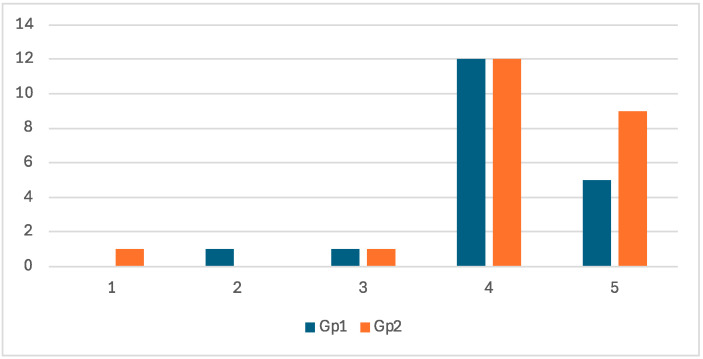
Likert scores for perceived realism of the simulator compared with reproductive tracts collected at the abattoir (n = 42).

Among experimental group trainees, 40.5% (n = 17) in Gp1 and 50.0% (n = 21) in Gp 2 considered the simulator realistic compared to abattoir specimens, totaling 90.5% positive responses (n = 38). However, 4.8% (n = 2) expressed neutral perceptions and 4.8% (n = 2) provided negative evaluations, equally distributed between groups. The mean Likert score was 4.17 ± 0.82 (median = 4) (Figure 1). In contrast, all experts (100%, n = 14) provided positive evaluations, with 57.1% (n = 8) scoring 5 points and 42.9% (n = 6) scoring 4 points, yielding a higher mean score of 4.57 ± 0.50 (median = 5).

When experimental groups compared the simulator to abattoir specimens, 85.7% (n = 36) considered it an exact replica [84.2% (n = 16) in Gp1 vs. 87.0% (n = 20) in Gp2], while 9.5% (n = 4) remained neutral (10.5% and 8.7% respectively) and 4.8% (n = 2) disagreed (one from each group). Comparing the simulator to live animals yielded similar results: 83.3% positive [n = 35, 15 (78.9%) in Gp1 and 20 (87.0%) in Gp2], 14.3% neutral [n = 6; 4 (21.1%) in Gp1 and 2 (8.7%) in Gp2], and 2.4% negative (n = 1; Gp2). Mean Likert scores were comparable: 4.07 ± 0.84 (median = 4) for abattoir specimens’ comparison and 4.10 ± 0.82 (median = 4) for live animal comparison. All experts (100%) agreed that simulator structures replicated those in cows, with 64.3% (n = 9) scoring 4 points and 35.7% (n = 5) scoring 5 points (mean: 4.36 ± 0.48, median = 4).

The simulator was perceived as more motivating than abattoir specimens by 90.5% of experimental group trainees [n = 38; 89.5% (n = 17) in Gp1, 91.3% (n = 21) in Gp2], while 7.1% (n = 3) held neutral opinions [2 in Gp1 and 1 in Gp2, 10.5 and 4.3%, respectively] and 2.4% (n = 1; Gp2) disagreed. The mean score was 4.29 ± 0.70 (median = 4). Similarly, 92.9% of experts (n = 13) found the simulator more motivating, with only 7.1% (n = 1) providing a negative rating (mean: 4.50 ± 0.82, median = 5).

#### 3.2.2. Physical Fidelity

**Figure 2:**
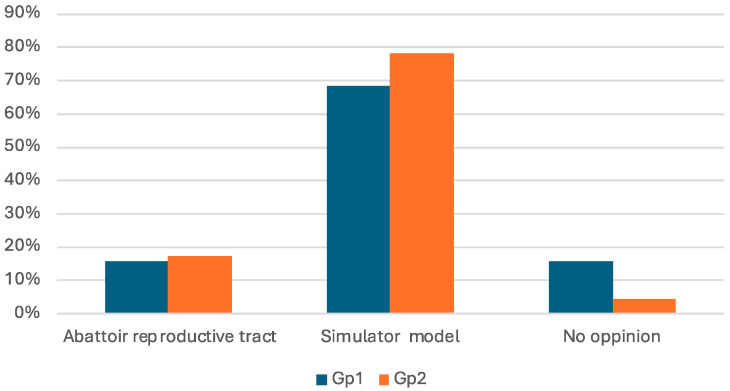
Trainees’ perceptions of the most realistic model (abattoir specimens vs. simulator model tract) compared to the living cow.

When asked which method better replicated working with live animals, 73.8% of experimental group trainees (n = 31) selected the simulator, 16.7% (n = 7) favored abattoir specimens, and 9.5% (n = 4) were unable to form an opinion. Figure 2 details the distribution of the respondents’ perceptions on which model was closer to the feeling of working in the living cow. Expert perspectives differed notably: 85.7% (n = 12) preferred a combination of both approaches, 14.3% (n = 2) selected simulator alone, and none chose abattoir specimens exclusively.

Trainee perceptions of TrAI4Nel simulator consistency were highly positive when compared to both abattoir specimens [95.2% positive (n = 40), 4.8% neutral or negative] and live animals [88.1% positive (n = 37), 11.9% neutral or negative]. Group-level analysis showed minimal variation: Gp1 vs. Gp2 positive responses were 94.7% vs. 95.6% for abattoir comparison and 84.2% vs. 91.3% for live animal comparison. Neutral responses were higher when comparing to live animals (10.5% in Gp1, 4.5% in Gp2). Mean scores were 4.24 ± 0.73 (median = 4) for abattoir comparison and 4.19 ± 0.87 (median = 4) for live animal comparison. Experts’ evaluations were uniformly positive: 57.1% (n = 8) scored 4 points, 42.9% (n = 6) scored 5 points (mean: 4.43 ± 0.50, median = 4).

**Figure 3:**
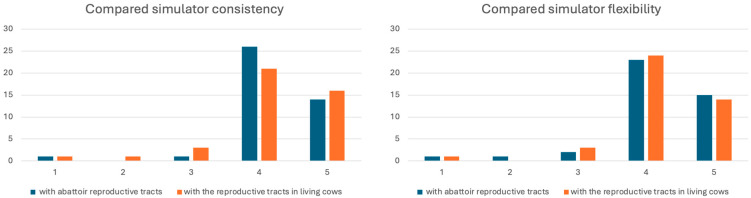
Likert scores for simulator model consistency (**right**) and flexibility (**left**) compared to reproductive tracts collected at the abattoir and in living cows.

Similar patterns emerged for flexibility evaluations. Overall positive responses above 90% were obtained for both abattoir and live animal comparisons, though group-level differences were noted: abattoir specimens comparison showed 94.7% positive in Gp1 vs. 87.0% in Gp2, while live animal comparison showed 84.2% vs. 95.7%, respectively. Negative perceptions were higher in Gp2 for abattoir specimens’ comparison (5.3% vs. 0% in Gp1), while neutral perceptions were higher in Gp1 for live animal comparison (15.8% vs. 0% in Gp2). Figure 3 details the score distributions for both parameters. Mean scores were 4.14 ± 0.89 (median = 4) for abattoir specimens’ comparison and 4.04 ± 0.93 (median = 4) for live animal comparison. Experts’ ratings were equally distributed between 4- and 5-point scores (50% each), yielding a mean of 4.50 ± 0.50 (median = 4.5).

The simulator’s ability to replicate procedural movements received positive evaluation from 92.9% of experimental group trainees (n = 39) [94.7% (n = 18) in Gp1, 91.3% (n = 21) in Gp2] when compared to abattoir specimens, with 4.8% neutral (n = 2) and 4.8% negative responses (n = 2), one from each group. Live animal comparisons yielded similar results: 90.5% positive [n = 38; 18 (94.7%) in Gp1 and 20 (87.0%) in Gp2], 4.8% neutral (n = 2; Gp2), and 4.8% negative (n = 2; Gp2). Mean scores were comparable: 4.26 ± 0.83 (median = 4) vs. 4.25 ± 0.95 (median = 4). Experts’ evaluations were consistently positive, with 57.1% (n = 8) scoring 4 points and 42.9% (n = 6) scoring 5 points (mean: 4.43 ± 0.50, median = 4).

#### 3.2.3. Content Validity

To establish the extent to which the simulator comprehensively covers all essential elements and aspects of the cattle artificial insemination procedure, questions used addressed two main domains: participants’ understanding of the need for using models and simulators in training, and their perceptions regarding whether the simulator accurately replicated key-steps of the insemination procedure (pipette passage through the cervix and identification of the semen deposition site).

Nearly universal agreement (98.4%, n = 60) was observed regarding the necessity of using anatomical models rather than direct training on live animals, with only one Gp2 participant (4.3%) disagreeing. Identical response patterns emerged for the importance of model practice before live procedures. Experts’ responses were more varied: 71.4% (n = 10) strongly agreed, 14.3% (n = 2) agreed moderately, while 14.3% (n = 2) strongly disagreed with model-based training necessity.

Overall, 95.1% of trainees (n = 58) reported that reproductive tract models enhanced their learning [94.7% (n = 18) GpC, 100% (n = 19) Gp1, 91.3% (n = 21) Gp2], while 3.3% (n = 2) in Gp2 disagreed and 1.6% (n = 1) in GpC expressed neutrality. For experimental groups specifically, the simulator’s learning contribution achieved a mean score of 4.71 ± 0.80 (median = 5).

The simulator demonstrated marked superiority over abattoir specimens in mimicking pipette passage through the cervix. All Gp1 participants (100%, n = 19) and 95.7% of Gp2 participants (n = 22) provided positive evaluations, with only 4.3% (n = 1) in Gp2 expressing neutrality (mean score: 4.36 ± 0.53, median = 4). Conversely, abattoir specimens received positive responses from only 57.1% of participants [n = 24; 68.4% (n = 13) Gp1, 47.8% (n = 11) Gp2], with 9.5% neutral (n = 4) and 33.3% negative responses (n = 14) [26.3% (n = 5) Gp1, 39.1% (n = 9) Gp2]. The mean score for abattoir specimens was significantly lower: 3.45 ± 1.25 (median = 4).

#### 3.2.4. Construct Validity

The simulator effectively facilitated skill transfer, with 95.2% of experimental group trainees (n = 40) indicating improved ability to replicate procedures in living cows [94.7% (n = 18) Gp1, 95.7% (n = 22) Gp2]. Only 2.4% expressed neutrality (n = 1, Gp2) and 2.4% disagreed (n = 1, Gp1). Mean score was 4.40 ± 0.66 (median = 4). Expert assessment was uniformly positive: 57.1% (n = 8) scored 4 points, 42.9% (n = 6) scored 5 points (mean: 4.57 ± 0.50, median = 5).

In contrast, GpC results with abattoir specimens showed limited effectiveness: only 31.6% (n = 6) found the training facilitated live animal procedures, while 52.6% (n = 10) experienced difficulties and 15.8% (n = 3) encountered some but surmountable challenges.

Simulator training strongly supported pipette navigation skills through the cervix, with 97.6% of experimental participants (n = 41) reporting improved pipette passage ability [100% (n = 19) Gp1, 95.7% (n = 22) Gp2], and only 2.4% (n = 1, Gp2) disagreeing. Mean score was 4.31 ± 0.55 (median = 4). All experts (100%) provided positive evaluations, distributed as 35.7% (n = 5) scoring 4 points and 64.3% (n = 9) scoring 5 points (mean: 4.64 ± 0.48, median = 5).

#### 3.2.5. Concurrent Validity

Direct comparisons revealed the simulator’s superiority over abattoir specimens across key parameters. For cervical navigation, 97.6% of experimental participants (n = 41) rated the simulator as closer to live cow experience [100% (n = 19) Gp1, 95.7% (n = 22) Gp2], with only 2.4% (n = 1, Gp2) expressing neutrality (mean: 4.36 ± 0.53, median = 4). Comparatively, abattoir specimens showed greater response variability: 57.1% positive [n = 24; 68.4% (n = 13) Gp1, 47.8% (n = 11) Gp2], 9.5% neutral [n = 4; 1 (5.3%) in GpC and three (8.7%) in Gp1], and 33.3% negative [n = 14; 26.3% (n = 5) Gp1, 39.1% (n = 9) Gp2] (mean: 3.50 ± 1.26, median = 4).

The simulator again outperformed abattoir specimens regarding the semen deposition site identification, with 92.9% of trainees (n = 39) providing positive ratings [94.7% (n = 18) Gp1, 91.3% (n = 21) Gp2], 2.4% neutral (n = 1, Gp1), and 4.8% negative (n = 2, Gp2) (mean: 4.38 ± 0.69, median = 4). Abattoir specimens received 64.3% positive responses [n = 27; 73.7% (n = 14) Gp1, 56.5% (n = 13) Gp2], 7.1% neutral (n = 3), and 28.6% negative (n = 12) [15.8% (n = 3) Gp1, 39.1% (n = 9) Gp2] (mean: 3.55 ± 1.13, median = 4).

**Figure 4:**
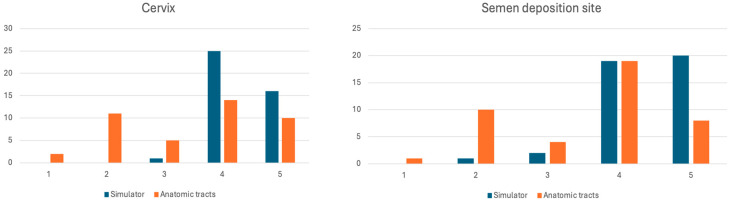
Likert scores for the perception of whether the training with reproductive tracts collected at the abattoir or the simulator provided a closer experience to the living cow concerning the passage of the cervix (**right**) and the identification of the semen deposition site (**left**).

Figure 4 presents details of the perceptions of trainees on these two parameters. The simulator consistently demonstrated reduced score dispersion compared to abattoir specimens across all measured parameters, indicating more reliable and predictable training experiences. This pattern was particularly evident in cervical navigation and semen deposition assessments, where abattoir specimens showed notably greater variability in participant responses.

Expert assessments were consistently favorable for both cervical passage [42.9% (n = 6) scoring 4 points, 57.1% (n = 8) scoring 5 points; mean: 4.57 ± 0.51, median = 5] and semen deposition site identification [28.6% (n = 4) scoring 4 points, 71.4% (n = 10) scoring 5 points; mean: 4.71 ± 0.47, median = 5].

#### 3.2.6. User Satisfaction

The simulator was perceived as easy to use by 39 (92.9%) trainees [19 (100%) in Gp1 and 20 (87.0%) in Gp2]. Only two trainees (4.8%) from Gp2 expressed a contrary opinion, while one participant (2.4%) from the same group maintained a neutral stance. All trainees (n = 42) in the experimental groups reported satisfaction with the simulator experience, with 13 participants scoring importance at 4 points and 29 at 5 points on the Likert scale. When asked about the simulator’s importance for AI training in cattle, 41 trainees (97.6%) agreed, with only one dissenting participant from Gp2. Similarly, all experimental group participants except one in Gp2 indicated that the simulator did not hinder their learning of cattle AI techniques.

Nearly all participants (97.6%) believed it was important to train first on the simulator before attempting the procedure on live cattle [19 (100%) in Gp1 and 22 (95.7%) in Gp2], with only one participant (2.4%) from Gp2 expressing disagreement.

All trainees agreed that simulator use provided them with greater autonomy when performing AI procedures on cattle [22 (52.4%) scored 4 points and 20 (47.6%) scored 5 points]. Similarly, all participants perceived the simulator as supporting their self-confidence for transferring the procedure to live animals [19 (45.2%) scored 4 points and 23 (54.8%) scored 5 points].

Regarding anxiety reduction, most trainees (39; 92.9%) expressed that simulator use helped reduce nervousness when performing cattle AI [18 (94.7%) in Gp1 and 21 (91.3%) in Gp2]. Two participants (4.8%) from Gp2 provided neutral responses, while one respondent (2.4%) from Gp1 expressed a negative response. The mean score for this item was 4.43 (SD = 0.70; median = 5). In addition, all but one trainee (97.6%) agreed that simulator use facilitated their learning, with the dissenting voice from Gp2.

Service providers evaluated the simulator’s potential contribution to trainee development. All experts agreed the simulator could enhance trainees’ autonomy and self-confidence [5 (35.7%) scored 4 points and 9 (64.3%) scored 5 points for both attributes], as well as reduce nervousness [6 (42.9%) scored 4 points and 8 (57.1%) scored 5 points] when first performing procedures on live cattle

The holistic content analysis of trainee responses was also undertaken to the open question “What contributed most to acquiring AI skills in cattle” revealed that 35 responses focused on the simulator. Six thematic categories emerged, namely, preparation and confidence, anatomy learning and anatomical localization, realism and fidelity to live animal, reduction of learning curve, technical aspects and handling, and animal welfare and ethical learning. Supportive quotations for each category are presented in Supplementary Materials Table S4. Several responses evidenced trainee appreciation for the progression from simulator to live animal. However, some perceived a few learning constraints emerged when comparing simulator training with abattoir specimens: “*when practicing with the pieces I didn’t feel difficulty, it was even easy, but on the simulator, it was more complicated*” [R11]. That was also evident from the analysis of responses regarding what contributed least to acquiring bovine AI skills; one participant identified a limitation: “*It was the ease of finding the rings in the simulator and in the cow there were a few more complications, but only at the beginning*” [R52].

Content analysis of experts’ feedback revealed five key themes regarding the simulator’s educational value and practical applications, most of which parallel those of the trainees: anatomical fidelity and realism, enhanced learning efficacy and skill development, animal welfare enhancement, professional confidence and preparedness, and innovative educational technology. Supplementary Materials Table S5 presents quotations for each category:

### 3.3. Training Effectiveness

The comparative analysis of training effectiveness revealed significant differences between simulator-based and traditional training approaches for artificial insemination technique acquisition.

#### 3.3.1. Training Sequence Comparison: “Simulator-First” vs. “Reproductive Tracts-First”

A direct comparison of the two experimental protocols (Table 4) provides critical insights into the role of the training sequence on skill acquisition. During the initial training phase, comprising attempts 1 and 2, the two experimental groups [Gp1—simulator-first (n = 19) and Gp2—reproductive tracts-first (n = 23)] demonstrated equivalent success rates in navigating the cow cervix (*p* = 0.664 for both attempts), suggesting that initial challenges are similar regardless of training sequence. In contrast, Gp2 significantly outperformed Gp1 during the third attempt (*p* = 0.037) (Table 4). Although overall training success increased compared with the two initial attempts (38.3% in Gp1 and 34.8% in Gp2; *p* = 0.449), final success rates were similar for both groups at the end of the training stage. Notably, the number of students who never succeeded in crossing the cow’s cervix was non-significantly higher in Gp2 (8.69%; n = 2) compared to Gp1 (n = 0). High overall success rates were observed in both groups at the final evaluation [84.2% in Gp1 vs. 73.9% in Gp2; *p* = 0.414] (Table 4). Collectively, these results indicate that training sequence does not significantly impact trainees’ final performance in groups that utilized the simulator.

A critical finding emerged when analyzing the number of attempts required to achieve overall success during training: successful participants in Gp2 required significantly fewer attempts to complete insemination (4.00 ± 0.74) compared to successful Gp1 trainees (7.95 ± 0.96 trials) (*p* < 0.001). This indicates that while Gp1 had a higher proportion of trainees who successfully completed the insemination procedure during training, they required more attempts to succeed compared to Gp2. This finding parallels the observation of a higher number of trainees who were unable to successfully navigate the cow’s cervix during the training stage in Gp1. The generalized eta-squared (η^2^G = 0.493) obtained for the number of attempts between experimental groups indicates that 49.3% of the variance was explained by the simulator order used in training. These findings support the hypothesis that initial exposure to biological specimens prior to simulator use enhances group performance efficiency and may facilitate skill development.

#### 3.3.2. Overall Success Rate Comparison: Control vs. Simulator Groups

The comparison between control (GpC; reproductive tract training only) and the groups that used the simulator (Gp1 and Gp2; simulator-based training) revealed contrasting performance patterns (Table 5). Although the control group trainees achieved higher success rates during the first training attempt (26.3 vs. 7.14%; *p* = 0.049), subsequent attempts showed no significant differences between groups. By the third trial, simulator groups showed numerical superiority (28.3% vs. 15.8%), although this difference did not reach statistical significance (*p* = 0.269). Overall training success rates were similar between control and experimental groups (37.5% vs. 36.5%, respectively; *p* = 0.724). Experimental groups required a slightly higher number of attempts until success during training (5.93 ± 0.65) compared with controls (5.29 ± 1.12) (*p* < 0.001), but the proportion of students who never succeeded in crossing the cervix with the AI catheter was similar between controls and experimental groups (*p* = 0.997; Table 5). The difference evidenced a large effect size (η^2^G = 0.721) in the number of attempts until first success during training.

Notably, simulator-trained students demonstrated significantly superior performance at final assessment, achieving 78.6% success compared to 52.6% in the control group (*p* = 0.043). This difference yielded a moderate effect size (η^2^G = 0.078) on the trainees’ success at the final examination.

#### 3.3.3. Detailed Three-Group Analysis

The comprehensive three-group comparison confirmed these findings with additional granularity. No differences existed between groups for first or second attempt performance (Table 6), despite slightly higher success percentages in the first attempt among GpC trainees (26.3% vs. 5.3% and 8.7% in Gp1 and Gp2, respectively). During the third attempt, Gp2 dramatically outperformed other groups [43.5% vs. 15.8% and 10.5% in GpC and GP2, respectively; *p* = 0.027]. Analysis of the number of attempts required for success revealed significant differences between groups. Gp2 participants required fewer attempts on average (4.00 ± 0.74) than both Gp1 (7.95 ± 0.96) and GpC (5.29 ± 1.12) to achieve success during the training phase (*p* < 0.001), indicating greater efficiency in Gp2 compared to the increased effort required for success in Gp1 and GpC. The estimated eta-squared (η^2^G = 0.365) indicates that 36.5% of the variance in the number of attempts resulted from the training method used. Conversely, Gp2 also presented the highest proportion of trainees who failed to successfully navigate the cervix during training (8.69%) compared with GpC (5.00%) and Gp1 (0.00%), although these differences did not reach statistical significance.

Most importantly, final assessment performance varied significantly among all three groups (*p* < 0.001), with Gp1 achieving the highest success rate (84.2%), followed by Gp2 (73.9%), and GpC showing the lowest performance (52.6%). This difference yielded a medium effect size (η^2^G = 0.078) on trainees’ success at the final examination.

Task completion time at the final assessment showed no significant differences between groups (*p* = 0.675), with mean completion times of 1.09 ± 0.72 min for Gp1, 1.30 ± 0.80 min for Gp2, and 1.33 ± 0.23 min for GpC. This suggests that improved success rates were achieved without increased time investment, indicating that simulator-enhanced training protocols improve learning efficiency rather than simply requiring more practice time.

## 4. Discussion

This study presents comprehensive validation of the TrAI4Nel simulator—a novel, anatomically precise mechanical simulator customized to train artificial insemination (AI) in Nelore cattle [17], demonstrating its effectiveness as an innovative educational tool for artificial insemination training in *Bos indicus* cattle. The validation encompassed multiple psychometric constructs—face, physical, content, construct, and concurrent validity—revealing consistently positive outcomes across all domains. The findings highlight the simulator’s effectiveness in supporting skill acquisition, improving trainee confidence, and providing a consistent and ethical alternative to traditional training methods, while addressing a critical gap in training resources specifically designed for zebu cattle reproductive anatomy.

### 4.1. Validation Construct Analysis

The high face validity scores (>4 points) from trainees and experts demonstrate that the TrAI4Nel successfully replicates the tactile and visual characteristics of zebu reproductive anatomy. This finding is particularly significant given the anatomical differences between *Bos indicus* and *Bos taurus* breeds, including narrower pelvic bones, longer cervices with firmer cervical rings, and deeper reproductive tracts [22]. The unanimous positive expert evaluation (100%) provides strong external validation of the simulator’s anatomical accuracy.

The physical validity results support the simulator’s realism, with 73.8% of trainees preferring it over abattoir specimens for replicating live animal conditions. Experts favored a combined approach (85.7%), suggesting that while the simulator provides superior standardization, integration with biological specimens may offer complementary benefits, aligning with educational theories advocating for multi-modal learning approaches [23]. The consistent superiority over abattoir specimens in flexibility and procedural movement replication addresses fundamental limitations of traditional training methods. Unlike abattoir specimens, which deteriorate over time and vary in quality, the simulator maintains consistent physical properties throughout extended use, providing standardized learning environments essential for large-scale training programs [23,24]. This standardization is especially valuable for competency-based assessment, where consistent evaluation criteria are essential for fair and reliable skill assessment.

The construct validity findings align with established simulation research demonstrating that high-fidelity simulators can enhance skill acquisition and retention [25,26]. However, effectiveness depends on factors beyond physical realism, such as progressive training methods and alignment with real-world tasks. Research has recommended focusing on functional fidelity relative to procedure demands rather than merely structural fidelity [27], though these aspects are interdependent. Low functional fidelity associates with longer time to acquire and transfer trained skills [28], while effectiveness varies with participant training levels [29], and progressive training from low to high fidelity models improves technical skill transfer [30].

The near-universal agreement (98.4%) regarding model-based training necessity before live animal procedures validates the educational rationale underlying simulator development, agreeing with established veterinary and animal sciences education principles emphasizing progressive skill development [24]. The simulator’s superiority in replicating critical procedural elements—particularly pipette passage through the cervix—demonstrates effectiveness in addressing the most challenging AI procedure aspects.

The embedded sensor and LED feedback system [17], represents a significant advancement over traditional methods, providing immediate corrective input that approaches self-regulated learning principles [31]. This real-time feedback addresses conventional training limitations where feedback often occurs after procedure completion, potentially reinforcing incorrect techniques.

Experimental group trainees reported increased self-confidence, greater procedural autonomy, and reduced anxiety when performing AI in cows, corroborated by superior success in final assessment accuracy and completion times compared to controls. Research shows high stress levels during invasive procedures negatively impact learning outcomes and animal welfare [32,33,34]. The simulator provides a psychologically safe environment facilitating early mistakes, experimentation, and confidence building without compromising ethical standards [35]. This preparedness for real-world application is critical for any training model, mirroring outcomes in high-fidelity simulation research across other domains [4,36,37]. Experts emphasized the simulator’s capacity to reproduce anatomical resistance, hand fatigue, and the tactile complexity of cervical structures—attributes essential to developing psychomotor skills.

Qualitative analysis revealed six key themes highlighting the simulator’s educational value: preparation and confidence building, anatomy learning, realism and fidelity, reduction of learning curve, technical skill development, and ethical learning considerations. These themes demonstrate that the simulator addresses multiple learning dimensions beyond technical skill acquisition. Animal welfare implications extend beyond immediate educational benefits, providing a standardized repeatable training that reduces live animals required while potentially improving care quality through better-prepared practitioners, aligning with replacement, reduction, and refinement principles [34].

The validated effectiveness holds particular significance for veterinary education programs, especially in regions where graduates will predominantly encounter *Bos indicus* cattle, and veterinary faculties in tropical and subtropical regions could substantially benefit from incorporating this specialized tool. The simulator enhances preparedness while reducing institutional dependence on abattoir specimens with their logistical challenges including procurement variability and biosafety management requirements.

### 4.2. The TrAIN4Nel Effectiveness for Translating Training to Practice

Training program effectiveness was confirmed through objective assessment of AI attempts in live animals. The most significant finding was simulator-trained students’ superior final assessment performance, achieving a 78.6% success rate compared to 52.6% in the control group—a 26-percentage-point improvement representing meaningful training effectiveness enhancement. The moderate effect size (η^2^G = 0.078) suggests simulator integration accounts for meaningful variance in final performance outcomes.

These results align with medical education principles where simulation-based learning consistently demonstrates superior outcomes across procedural skills programs. Our findings are noteworthy compared to similar studies. Dalton et al. [38] reported 22-percentage-point increases in post-test knowledge scores following AI training using abattoir reproductive tracts but focused on theoretical knowledge rather than practical application. Contrasting with that study, developed with veterinary medicine students, our study demonstrates practical skill transfer with 26-percentage-point improvement in actual procedural success rates in trainees without former knowledge in bovine anatomy, suggesting particular effectiveness for hands-on skill development.

The SIMCA-COW validation study showed significant improvement between first and fourth training attempts [39], consistent with our observations of progressive skill development. However, unlike studies focusing primarily on training progression, our research provides comprehensive evidence of superior final assessment performance compared to traditional methods, establishing practical relevance and supporting TrAIN4Nel’s ability to foster competency translation to real scenarios.

Enhanced performance likely stems from the simulator’s ability to provide standardized, repeatable training experiences allowing muscle memory and procedural confidence development in controlled environments before progressing to live procedures. Recent reviews note that models and simulators reduce animal stress while decreasing student anxiety and improving motivation, self-confidence, and self-efficacy [32,33,34,39], factors that likely contribute to superior learning outcomes.

Our experimental design enabled comparative training sequence analysis, revealing nuanced learning efficiency differences. While both experimental groups achieved similar final success rates whether using a simulator-first (Gp1) or reproductive tracts-first approach (Gp2), the latter demonstrated superior learning efficiency during training. Successful Gp2 participants required significantly fewer attempts, with training sequence explaining 49.3% of variance in attempts required (η^2^G = 0.493). This suggests initial biological specimens’ exposure before simulator training might enhance efficiency by providing authentic tactile feedback and anatomical variability that improves simulator skill translation ability. Our results complement other simulator validation studies while providing novel training sequence optimization insights. Azuaga Filho et al. [11] noted traditional genital tract models from slaughterhouses present disadvantages including tissue tension loss and decreased reliability after repeated use. Our study confirms lower control group success rates and suggests strategic biological specimens’ integration with simulator training optimizes learning efficiency while maintaining both approaches’ benefits. This sequence potential accelerates learning curves by improving pattern recognition and procedural adaptation when transitioning to simulator-based practice.

The observation that task completion times showed no significant differences between groups (*p* = 0.675) despite improved success rates indicates simulator-enhanced training protocols improve learning effectiveness rather than requiring extended practice time. Higher success rates with equivalent completion times suggests simulator training enhances procedural accuracy and decision-making efficiency—core competencies essential for successful artificial insemination practice.

Superior skill transfer addresses a critical literature concern regarding simulator-to-live animal transition difficulties. Previous studies with commercial simulators like Breed’n Betsy and Henryetta have reported general difficulties when trainees progress from models to live cows. Our integrated approach appears to mitigate transition challenges by providing both authentic tissue experience and standardized simulator practice.

Initially higher control group success rates during first training attempts (26.3% vs. 7.14%; *p* = 0.040) merit consideration. This early advantage likely reflects biological specimen training’s immediate authenticity, where students encounter real structures from the outset, agreeing with Dalton et al. [38]. However, this initial advantage was not sustained and was ultimately overcome by superior long-term simulator-trained group performance, suggesting traditional methods may provide immediate familiarity but lack the systematic skill-building advantages offered by simulator technology.

## 5. Study Limitations and Future Directions

While our findings provide strong support for simulator-based training, several limitations warrant consideration. The study population was limited to bovine AI provider candidates without previous knowledge in bovine anatomy or reproduction, potentially introducing uncontrolled variability in baseline skill levels and learning curves. The absence of confirmatory diagnostic techniques (e.g., ultrasound, dye tracking, post-mortem verification) to objectively determine pipette placement accuracy limits skill transfer assessment precision. Future studies should integrate these objective endpoints and expand evaluation to include long-term retention and field performance metrics.

The inconclusive results regarding optimal simulator presentation order (before or after abattoir specimen training) warrant additional investigation, as this has important curriculum design implications and supports progressive fidelity training concepts proposed by Brydges et al. [30]. The data suggest that optimal training sequences might involve initial biological variability exposure followed by standardized simulator practice, maximizing both training modalities’ benefits while minimizing individual limitations.

Our findings address critical gaps identified in recent literature reviews, considering that validation of available commercial simulators for AI training remains insufficient, with most results relying on user perception rather than objective measures [11]. Our study provides objective performance measures and statistical validation, moving beyond subjective face validity assessments characterizing the existing literature. Future investigations should examine the cost-effectiveness of different simulator implementation strategies and explore hybrid training approaches optimizing both biological specimen exposure and simulator-based practice.

Integration of advanced technologies—including sensors for electronic or electromagnetic feedback, virtual reality enhancements, and artificial intelligence methods for technique validation—represents a promising direction for addressing current limitations in objective skill assessment and providing more comprehensive training evaluation capabilities.

## 6. Conclusions

This study provides robust evidence that simulator-based training significantly enhances artificial insemination skill acquisition and practical application success. Both simulator-based training sequences outperformed traditional methods in final competency assessment, with reproductive tracts-first approaches demonstrating superior learning efficiency. These findings support integrating simulator technology into animal reproduction education curricula.

The TrAI4Nel simulator represents a significant advancement in bovine reproductive training, offering an ethically responsible, cost-effective, and technically accurate alternative to traditional AI training models. Its comprehensive validation, strong user acceptance, and proven practical impact support integration into AI training curricula for *Bos indicus* cattle. Superior performance with equivalent task completion times demonstrates enhanced learning quality rather than extended practice requirements.

The simulator’s scalability, reproducibility, and scenario simulation capabilities make it particularly attractive for structured training programs across regions where *Bos indicus* breeds predominate, positioning simulation technology as increasingly indispensable for ethical, effective skill development.

## Figures and Tables

**Table 1:** Age of participants in each trainees’ group and experts.

*Participants*	*Groups*	*n*	*Mean ± SD*	*Median*	*Min*	*Max*
*Trainees*	Gp C	19	28.32 ± 2.72	26	16	53
	Gp 1	19	23.16 ± 1.50	20	17	39
	Gp 2	23	28.87 ± 2.19	27	17	54
*Experts*		14	34.43 ± 11.27	29	24	62

**Table 2:** Educational background of the trainees.

*Groups*	*Education Level*	*Frequency*	*Percentage*
*Gp C (n = 19)*	Grade school	1	5.26
	High school	15	78.95
	College incomplete	1	5.26
	College complete	2	10.53
*Gp 1 (n = 19)*	Grade school	0	0.00
	High school	14	73.68
	College incomplete	1	5.26
	College complete	4	21.05
*Gp 2 (n = 23)*	Grade school	0	0.00
	High school	11	47.83
	College incomplete	4	17.39
	College complete	8	34.78

**Table 3:** Occupational activities of the trainees.

*Occupational Activities*	*Groups*			
	GpC (n, %)	Gp1 (n, %)	Gp2 (n, %)	Total
*Unemployed*	2 (10.53%)	5 (26.32%)	1 (4.35%)	8
*Self-employed*	1 (5.26%)	1 (5.26%)	4 (17.39%)	6
*Administrative assistant*	1 (5.26%)	2 (10.53%)	6 (26.09%)	9
*Entrepreneur*	1 (5.26%)	0 (0.00%)	3 (13.04%)	4
*Student*	5 (26.32%)	3 (15.79%)	4 (17.39%)	12
*Livestock worker/cattle herder*	2 (10.53%)	2 (10.53%)	1 (4.35%)	5
*Rural producer/Farmer*	6 (31.58%)	5 (26.32%)	1 (4.35%)	12
*Public servant*	1 (5.26%)	1 (5.26%)	0 (0.00%)	2
*Homemakers*	0 (0.00%)	0 (0.00%)	2 (8.70%)	2
*Zootechnician*	0 (0.00%)	0 (0.00%)	1 (4.35%)	1
*Totals*	19	19	23	61

**Table 4:** Effects of training sequence on success (% of trainees who successfully navigated the cow cervix) during cow insemination procedure in experimental groups [simulator-reproductive tract (Gp1; n = 19) vs. reproductive tracts-simulator (Gp2; n = 23)].

Success Assessment	Gp1	Gp2	*p*-Value
Attempt 1 (%; counts:n)	5.3 (1:19)	8.7 (2:23)	0.664
Attempt 2 (%; counts:n)	5.3 (1:19)	8.7 (2:23)	0.664
Attempt 3 (%; counts:n)	10.5 (2:19)	43.5 (10:23)	**0.037**
Mean number of attempts until success during training (±SEM) [min; max]	7.95 ± 0.96 [1, 15]	4.00 ± 0.74 [1, 13]	**<0.001**
Never successful during training (%; counts:n)	0.00 (0:19)	8.69 (2:23)	0.191
Mean overall success during training attempts	38.3 (134:350)	34.8 (128:368)	0.449
Assessment success	84.2 (16:19)	73.9 (17:23)	0.414

**Table 5:** Percentage of success at cow insemination procedure in experimental groups [reproductive tract only (GpC; n = 19) vs. simulator (Gp1 and Gp2; n = 42)].

Successful Assessment (%)	GpC	Experimental Groups (Gp1 and Gp2)	*p*-Value
Attempt 1 (%; counts:n)	26.3 (5:19)	7.14 (3:42)	**0.040**
Attempt 2 (%; counts:n)	8.7 (2:19)	7.14 (3:42)	0.662
Attempt 3 (%; counts:n)	15.8 (3:19)	28.6 (12:42)	0.269
Mean number of attempts until success during training (±SEM) [min; max]	5.29 ± 1.12 [1, 17]	5.93 ± 0.65 [1, 15]	**<0.001**
Never successful during training (%; counts:n)	5.00 (1:19)	4.76 (2:42)	0.997
Mean overall success during training attempts	37.5 (161:429)	36.5 (262/718)	0.724
Assessment success	52.6 (10:19)	78.6 (33:42)	**0.043**

**Table 6:** Three-group comparison for overall success at cow insemination procedure and in each of the first three attempts in control (GpC; n = 19) and experimental groups [simulator-reproductive tract (Gp1; n = 19) vs. reproductive tracts-simulator (Gp2; n = 23)].

Success Assessment	GpC	Gp1	Gp2	*p*-Value
Attempt 1 (%; counts:n)	26.3 (5:19)	5.3 (1:19)	8.7 (2:23)	0.130
Attempt 2 (%; counts:n)	8.7 (2:19)	5.3 (1:19)	8.7 (2:23)	0.830
Attempt 3 (%; counts:n)	15.8 (3:19) ^a^	10.5 (2:19) ^a^	43.5 (10:19) ^b^	**0.027**
Mean number of attempts until success during training (±SEM) [min; max]	5.29 ± 1.12 ^a^ [1, 17]	7.95 ± 0.96 ^b^ [1, 15]	4.00 ± 0.74 ^c^ [1, 13]	**<0.001**
Never successful during training (%; counts:n)	5.00 (1:19)	0.00 (0:19)	8.69 (2:23)	0.430
Mean overall success during training attempts	37.5 (161:429)	38.3 (134:350)	34.8 (128:368)	0.586
Assessment outcome/success	52.6 (10:19) ^a^	84.2 (16:19) ^b^	73.9 (17:23) ^c^	**<0.001**

Different superscript letters indicate statistical significance at post hoc test.

## Data Availability

The raw data supporting the conclusions of this article are available from the corresponding author, upon reasonable request and ethical compliance.

## Supplementary Materials

The following supporting information can be downloaded at: https://www.mdpi.com/article/10.3390/ani15202982/s1, Table S1: Construct of the questionnaire used to collect trainees’ perceptions; Table S2: Construct of the questionnaire used to collect professionals’ perceptions; Table S3: Allocation of the questionnaire items to each validation constructs; Table S4: Thematic categories emerging from the trainees’ perceptions on the simulator; Table S5: Thematic categories emerging from the experts’ perceptions on the simulator; Figure S1: Overview of the simulator’s main features, illustrating its key components and functionalities.
