# Supplementary material for: Comprehensive Validation of the TrAI4Nel Simulator for Nelore Artificial Insemination Training: A Controlled Study
Source: Animals (Basel). 2025 Oct 15;15(20):2982. doi: 10.3390/ani15202982 (PMC12560861; doi:10.3390/ani15202982)
Supplement: Supplementary file 1 [file animals-15-02982-s001.zip › Supplementary tables.pdf]

Supplementary table S1– Construct of the questionnaire used to collect trainees' perceptions

**Section 1: Informed Consent**	yes/no [precluded following to the questionnaire if NO was selected]	
**Section 2: Personal Information**	Email [to allow pairing with AI assessment]	Open Question
	Gender	Female/Male/Prefer not to say
	Age (in years)	numeric
	Education level	Open Question
	What is your occupation?	Open Question
	Do you have experience with livestock?	Yes/No
	Are you familiar with artificial insemination in cattle?	Yes/No
	Have you ever inseminated a cow?	Yes/No
**Section 3: Training Experience**		
1. Identification of experimental groups	In my group, the training took place:	Without | With a simulator (go to section 4)
2. Use of biological models (reproductive tracts) during the course:	• Sufficient information was provided at the start of the course about the proposed learning method.<br>• I fully understood the objectives of using biological models (reproductive tracts) for learning.<br>• I consider it important to practice procedures on models before performing them on live animals.<br>• Using models facilitated learning the technique.	Likert scale
3. In comparison to live cattle examination, the biological models of reproductive tracts	• Help me recognize the shape and consistency of reproductive structures encountered in the cow<br>• Facilitated replication of procedures when working with live cattle<br>• Allowed me to be more careful in handling with live animal.<br>• The structures of the biological model used were similar to those found in the animal<br>• Facilitated passage of the insemination pipette in the live animal<br>• Facilitated learning of the technique<br>• Demonstrated anatomical structures appropriate for the required competency level<br>• The structures of the reproductive tracts were similar to those of living cows<br>• Increased my confidence in performing procedures on live animals	Likert scale

	• Maintained motivation throughout the learning process	
4. Transitioning from biological models (reproductive tracts) to the cow	• It was easy.<br>• I had some difficulty, but I overcame it quickly.<br>• I still found it difficult to identify the reproductive tract	Multiple choice
5. For comparison, the procedure of AI pipette passage in the live animal	• was harder than in the biological model.<br>• was about the same difficulty as in the biological model.<br>• was easier than in the biological model.	Justify your answer
**End of the questionnaire for the control group**		
**Section 4: Simulator Evaluation**		
1. Regarding the use of simulator during the course:	• Sufficient information was provided at the start about the learning method.<br>• I fully understood the objectives of using models/simulators for learning.<br>• I consider it important to practice procedures on models and simulators before performing them on live animals.<br>• Using models and simulators did not hinder my learning.<br>• Using models and simulators helped my learning.	Likert scale
2. In my course:	• First, I worked on biological models, then the simulator, and only then on the cow.<br>• First, I worked on the simulator, then on biological models, and only then on the cow.	Multiple choice
3. Think about how you felt training on abattoir reproductive tracts and the simulator:	• The simulator was very realistic.<br>• In terms of consistency, the structures were similar to those found in the abattoir pieces.<br>• In terms of consistency, the structures were similar to the real cow.<br>• In terms of flexibility, the structures were similar to those found in the abattoir pieces.<br>• In terms of flexibility, the structures were similar to the real cow.<br>• The simulator was an exact replica of the abattoir reproductive tracts<br>• The simulator was an exact replica of those found in a real cow<br>• I could manipulate (repeat basic movements) as well on the simulator as on the pieces.<br>• I could manipulate (repeat basic movements) as well on the simulator as on the cow.	Likert scale

4. Between the abattoir tracts and the simulator, which was more motivating for training the procedures	• Biological models (reproductive tracts)<br>• Simulator<br>• No opinion	Multiple choice
5. Between the abattoir pieces and the simulator, which better reflected reality?	• Biological models (reproductive tracts)<br>• Simulator<br>• No opinion	Multiple choice
6. Compare your training experience in AI using abattoir models or the simulator to the living cow	• For passing the pipette through the cervix t the abattoir reproductive tracts were closer to the cow<br>• For passing the pipette through the cervix the simulator was closer to the cow<br>• For detecting the semen deposition site, the abattoir reproductive tracts were closer to the cow<br>• For detecting the semen deposition site, the simulator was closer to the cow	Likert scale
7. Provide your opinion on the use of the simulator	• The simulator was easy to use.<br>• It gave me more autonomy in the procedure.<br>• It increased my confidence in performing the procedure.<br>• It facilitated passing the pipette through the cervix.<br>• It facilitated performing the procedures on the animal.<br>• It was more stimulating for technical training.<br>• It better highlighted aspects to consider when working with live animals.<br>• It was more stimulating for technical training.<br>• It helped reduce nervousness	Likert scale
**Section 5: Final Considerations**	How satisfied are you with the use of the simulator?	Likert scale
	How important was the simulator for procedure training?	Likert scale
	Was the course beneficial to you?	Yes/No
	At the end of the course, do you feel confident to perform AI in cattle independently?	Yes/No
	What contributed the most to acquiring AI skills in cattle?	Open Question
	What contributed the least to acquiring AI skills in cattle?	Open Question

Supplementary table S2– Construct of the questionnaire used to collect professionals' perceptions

**Section 1: Informed Consent**	yes/no [precluded following to the questionnaire if NO was selected]	
**Section 2: Personal Information**	Email [to allow pairing with AI assessment]	Open Question
	Gender	Female/Male/Prefer not to say
	Age (in years)	numeric
	Are you familiar with artificial insemination in bovines?	Open Question
	How many years have you been performing artificial insemination in bovines?	Open Question
	Do you perform artificial insemination in bovines regularly or sporadically?	Open Question
	Have you ever experimented with or utilized an artificial insemination simulation model?	Yes/No
	If you answered yes to the previous question, please indicate which model you used.	Open question
**Section 2: Simulator Evaluation**		
1. Regarding the use of simulator during the course:	• Sufficient information was provided at the start about the learning method.<br>• I fully understood the objectives of using simulators/simulators for learning.<br>• I consider it important to practice procedures on models and simulators before performing them on live animals.<br>• I consider that the use of models and simulators does not harm learning<br>• I consider that the use of simulators has potential to facilitate learning	Likert scale

2. Think about the sensation you have when performing insemination on a live animal versus on the simulator	• The simulator was very realistic.<br>• In terms of consistency, it was similar to that found in a real cow<br>• In terms of flexibility, it was similar to that found in a real cow<br>• It was an exact replica of those found in a real cow<br>• I was able to manipulate (repeat basic movements) as well on the simulator as on a real cow<br>• The simulator would be more motivating for trainees to practice procedures than abattoir specimens<br>• The simulator is more useful than abattoir specimens for identifying structures important for AI (artificial insemination)	Likert scale
3. Between abattoir specimens and the simulator, which do you think better facilitates training of procedures and their replication in animals?	• Biological models (reproductive tracts)<br>• Simulator<br>• No opinion	Multiple choice
4. Between abattoir specimens used traditionally and the simulator, which do you consider better reflects the reality found in the animal?	• Biological models (reproductive tracts)<br>• Simulator<br>• A combination of both<br>• No opinion	Multiple choice
5. Comparing the simulator and the real animal	• The simulator was very close to the reality of the cow for cervical passage<br>• The simulator was very close to the reality of the cow for identifying semen deposition sites	Likert scale
6. Between abattoir specimens and the simulator, which do you consider will be more useful in technique training?	• Biological models (reproductive tracts)<br>• Simulator<br>• No opinion	Multiple choice
7. Compared to traditional use of specimens, the simulator will have the capacity to:	• Acquire more autonomy in the procedure<br>• Acquire greater confidence in the procedure to be performed<br>• Facilitate passage of the pipette through the cervix	Likert scale
8. Compared to traditional use of specimens, the simulator will have the capacity to:	• Facilitate performance of procedures on the animal<br>• Be more stimulating for technique training<br>• Better draw attention to aspects to consider when working with live animals<br>• Allow reduction of nervousness when working with live animals	
**Section 3: Final Considerations**		

1. Potential importance of the simulator in procedure training	• Do you find the simulator useful in training bovine AI technique?<br>• Do you think it's important to use the simulator before testing the technique on cows?	Likert scale
	Globally, what is your level of satisfaction regarding the use of the simulator?	Open Question

Supplementary table S3– Allocation of the questionnaire items to each validation constructs

Validity type	Questionnaire version	items
**Face Validity**	Trainees' questionnaire	Section 3.3 The structures of the reproductive tracts were similar to those of living cows<br>Section 4.3 The simulator was very realistic<br>Section 4.3 The simulator was an exact replica of the abattoir reproductive tracts<br>Section 4.3 The simulator was an exact replica of those found in a real cow<br>Section 4.4. Between the abattoir tracts and the simulator, which was more motivating for training the procedures
	Professionals' questionnaire	Section 2.2 The simulator was very realistic<br>Section 2.2 The simulator was an exact replica of those found in a real cow<br>Section 2.2. The simulator would be more motivating for trainees to practice procedures than abattoir specimens
**Physical fidelity**	Trainees' questionnaire	Section 4.5 Between the abattoir pieces and the simulator, which better reflected reality?<br>Section 4.3 In terms of consistency, the structures were similar to those found in the abattoir pieces.<br>Section 4.3 In terms of consistency, the structures were similar to the real cow.<br>Section 4.3 In terms of flexibility, the structures were similar to those found in the abattoir pieces.<br>Section 4.3 In terms of flexibility, the structures were similar to the real cow.<br>Section 4.3 I could manipulate (repeat basic movements) as well on the simulator as on the pieces<br>Section 4.3 I could manipulate (repeat basic movements) as well on the simulator as on the cow.<br>Section 4.5. Between the abattoir pieces and the simulator, which better reflected reality?
	Professionals' questionnaire	Section 2.2. In terms of consistency, it was similar to that found in a real cow<br>Section 2.2. In terms of flexibility, it was similar to that found in a real cow<br>Section 2.2. I was able to manipulate (repeat basic movements) as well on the simulator as on a real cow<br>Section 2.4. Between abattoir specimens traditionally used and the simulator, which do you consider better reflects the reality found in the animal?
**Content Validity**	Trainees' questionnaire	Section 3.2 & 4.1 I fully understood the objectives of using simulators for learning<br>Section 3.2 & 4.1 I consider it important to practice procedures on models before performing them on live animals.<br>Section 3.2 Using models facilitated learning of the technique & 4.1 Using models and simulators helped my learning<br>Section 3.3 Facilitated passage of the insemination pipette in the live animal<br>Section 4.7 It facilitated passing the pipette through the cervix.
	Professionals' questionnaire	Section 1.1 I fully understood the objectives of using simulators/simulators for learning<br>Section 2.7 Facilitate passage of the pipette through the cervix

**Construct Validity**	Trainees' questionnaire	Section 4.7 It facilitated passing the pipette through the cervix.<br>Section 4.7 It facilitated performing the procedures on the animal.<br>Section 5 At the end of the course, do you feel confident to perform AI in cattle independently?
	Professionals' questionnaire	Section 2.7 Facilitate passage of the pipette through the cervix<br>Section 2.8 Facilitate performance of procedures on the animal
**Concurrent Validity**	Trainees' questionnaire	Section 4.6 For passing the pipette through the cervix the abattoir reproductive tracts were closer to the cow<br>Section 4.6 For passing the pipette through the cervix the simulator was closer to the cow<br>Section 4.6 For detecting the semen deposition site the abattoir reproductive tracts were closer to the cow<br>Section 4.6 For detecting the semen deposition site the simulator was closer to the cow
	Professionals' questionnaire	Section 2.2 The simulator is more useful than abattoir specimens for identifying structures important for AI (artificial insemination)<br>Section 2.5 The simulator was very close to the reality of the cow for cervical passage<br>Section 2.5 The simulator was very close to the reality of the cow for identifying semen deposition sites
**Users'Feedback**	Trainees' questionnaire	Section 4.7 The simulator was easy to use<br>Section 4.7 It gave me more autonomy in the procedure<br>Section 4.7 It increased my confidence in performing the procedure<br>Section 4.7 It helped reduce nervousness<br>Section 5 What contributed the most to acquiring AI skills in cattle?<br>Section 5 What contributed the least to acquiring AI skills in cattle?
	Professionals' questionnaire	Section 2.7 Acquire more autonomy in the procedure<br>Section 2.7 Acquire greater confidence in the procedure to be performed<br>Section 2.8 Allow reduction of nervousness when working with live animals<br>Section 3.1 Globally, what is your level of satisfaction regarding the use of the simulator?

Supplementary table S4. Thematic categories emerging from the trainees' perceptions on the simulator

Thematic categories	Interpretation	Quotations
Preparation and Confidence	Trainees emphasized the simulator's fundamental preparatory role	"*Preparation by the simulator before the cows*" [R19]<br>"*The simulator helped a lot, without the simulator the experience wouldn't be the same, I went confident to inseminate*" [R18]<br>"*It was having done the activities on the simulator [...] having gone through the simulator to the cows with more confidence in what I'm doing*" [R17]<br>"*The simulator certainly served to help us and give us an idea of what it would be like before having contact with the animal*" [R51]
Anatomy Learning and Anatomical Localization	The simulator's importance for anatomical understanding was highlighted	"*Training skills in locating the cervix*" [R08]<br>"*The simulator! Because from it we had much facility in identifying the reproductive apparatus in the live animal*" [R35]<br>"[...] *especially the simulator helped me a lot to identify the cervix, the vagina in the animal*" [R60]<br>"*To improve knowledge, I believe the simulator helped a lot in knowledge of the anatomical area itself*" [R50]
Realism and Fidelity to Live Animal	The similarity between simulator and real conditions was valued	*"Now on the simulator it was more complicated, it's more similar to the live cow because on the simulator the arm gets very tight just like in the cow*" [R11]<br>"*The exercise on the simulator in practice was close to the characteristics produced by the animal*" [R10]<br>"*The mannequin, because the similarity to the live animal is alike*" [R33]<br>" [...] *the use of the simulator, which looks very much like the animals themselves*" [R31]
Reduction of Learning Curve	Evidence that the simulator facilitated transition to live animals	"*For those who had no experience with cows, the simulator helped a lot*" [R09]<br>"*The use of cow simulators, as it facilitated procedures on the live animal!*" [R32]<br>*"[…]the mannequin simulates the reality of the tightness inside the cow, the distance where we can find the cervix, simulates the entry orifice where the pipette should pass and the cervical rings where we have to find the target, and it doesn't contaminate us since it's not biological material. The mannequin also blocks our vision, helping us in the part of seeing the piece through imagination, which also happens in the cow"* [R34]<br>"*For me what contributed most were the ´artificial cows` because they gave me a foundation of what it would be like in a real animal*" [R48]

Technical Aspects and Handling	Facilitated the development of specific technical competencies	“[overcoming] t*he difficulty of passing the pipette in the simulator*" [R07]<br>"*First of all was using the simulator, because with it I had an opening in knowledge that contributed a lot for me to learn to do the right handling*" [R22]<br>"*The simulator helped me a lot [...] how I should handle the pipette to find the target*" [R60]
Animal Welfare and Ethical Learning	Recognition of the importance of prior practice with simulator to minimize animal stress	“*When performed on the artificial piece there's a chance to learn without hurting the animal, improving technique on the plastic piece*" [R34]<br>"*Having a model to train, before going to the live animal, helped me overcome my fears of handling an animal*" [R58]

Supplementary table S5. Thematic categories emerging from the experts' perceptions on the simulator

Thematic categories	Interpretation	Quotations
Anatomical Fidelity and Realism	Exceptional anatomical accuracy was emphasized	*"…quite similar to the live animal, especially regarding the cervical rings and the weight of the reproductive apparatus (…) sensation of hand fatigue when extensively manipulated*" [P05]<br>"*… the simulator perfectly represents the reproductive structures, size, texture, weigh (…) the difficulties encountered with the simulator are identical to reality" [P03]*<br>" *… super realistic and well-detailed, able to demonstrate different cervical sizes and shapes* " [P13]<br>*"Simulator very similar to the animal but with less sensitivity"* [P11]
Enhanced Learning Efficacy and Skill Development	The simulator may improve learning outcomes	*"…with a student training on the simulator, he will have faster learning*" [P07]<br>"*… the simulator reduces errors in hitting the target because it indicates when you are in the right location. This is very important (…) it prepares the student for what he will face inside the animal* " [P12]
Animal Welfare Enhancement	Emphasis on reduced reliance on live animals	*"…very important, because it is very similar to the animal and with this reduces the use of training on animals*" [P04]<br>"*… will contribute to animal welfare, since the student will be more familiar with the correct technique, avoiding inappropriate movements that harm the animal*" [P03]<br>"*… will bring better practicality in relation to training due to not causing discomfort to animals, providing better welfare to animals with the professional already trained to perform the technique practice*" [P08]
Professional Confidence and Preparedness	Impact on trainee readiness	*"…an innovative, necessary and important experience, because it portrays very well the reproductive system of a cow. It provides security and an idea of how it will be in practice with the live animal*" [P06]<br>" *… adding much value for the professional who will perform the technique on a live animal*" [P11]
Innovative Educational Technology	Appreciation for technological features	*"…the simulator was incredible, the anatomical difference of the structures caught my attention, it brings to us the differences that can be found in animals*" [P14]<br>" *… the peristalsis simulation was also excellent, portraying one of the challenges encountered during an insemination*" [P01]
