# Supplementary figures and images for: Comprehensive Validation of the TrAI4Nel Simulator for Nelore Artificial Insemination Training: A Controlled Study
Source: Animals (Basel). 2025 Oct 15;15(20):2982. doi: 10.3390/ani15202982 (PMC12560861; doi:10.3390/ani15202982)

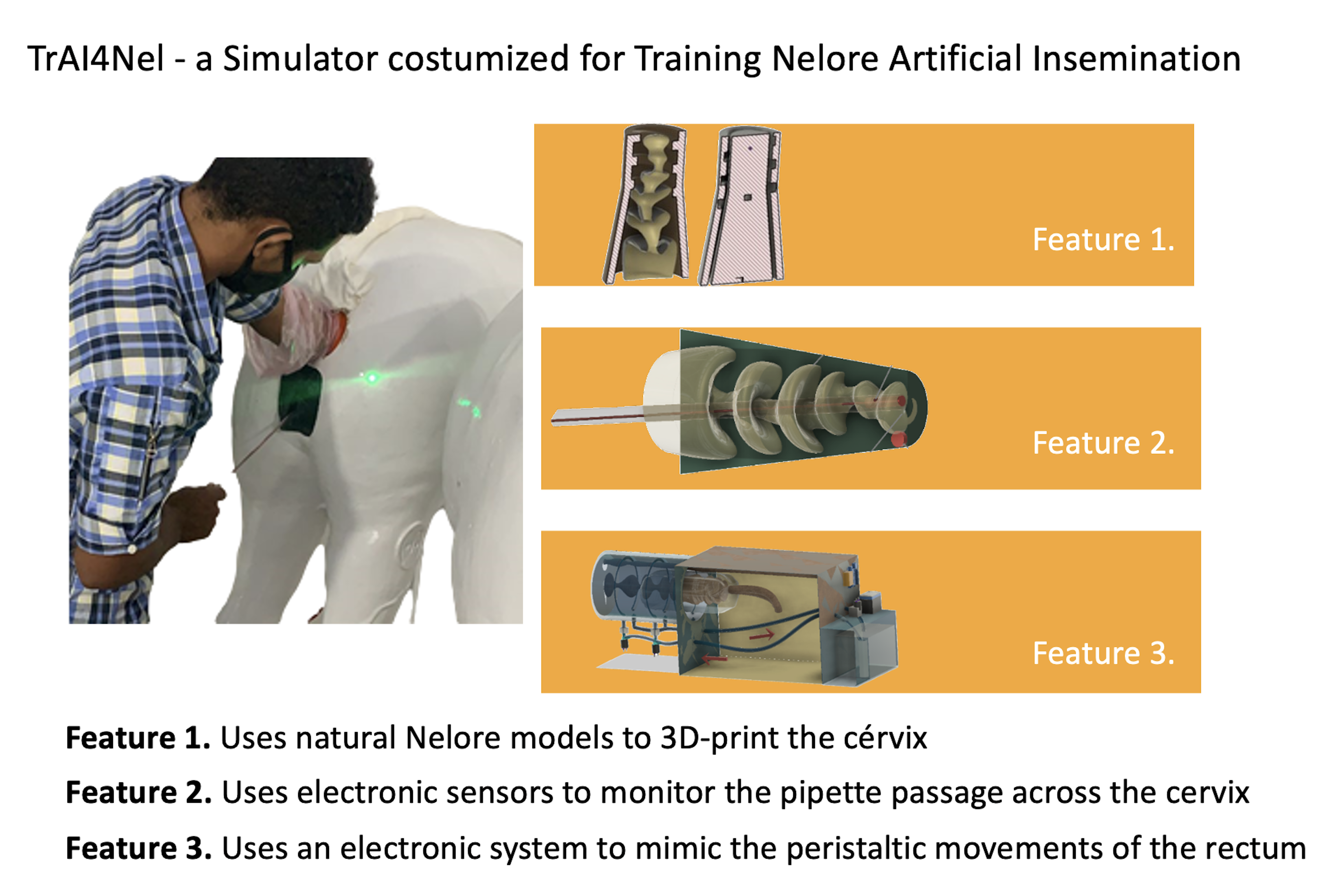

Supplement: Supplementary file 1 [file animals-15-02982-s001.zip › Supplementary Figure S1.png]
